# Placebo hypoalgesia induced by operant conditioning: a comparative study on the effects of verbal, token-based, and social rewards and punishers

**DOI:** 10.1038/s41598-023-47482-1

**Published:** 2023-11-21

**Authors:** Helena Bieniek, Przemysław Bąbel

**Affiliations:** 1 Institute of Psychology, Pain Research Group, Jagiellonian University, ul. Ingardena 6, 30-060 Kraków, Poland; 2 Doctoral School in the Social Sciences, Jagiellonian University, Kraków, Poland

**Keywords:** Human behaviour, Placebo effect

## Abstract

Operant conditioning was shown to be a mechanism of placebo hypoalgesia; however, only verbal rewards and punishers were applied in the previous study. We aimed to induce placebo hypoalgesia using more clinically relevant consequences: token-based and social. Participants were divided into three experimental groups (with verbal, social, and token-based rewards and punishers); and two control groups (with and without placebo application). During operant conditioning, participants in the experimental groups received thermal stimuli of equal intensity and were rewarded for reporting lower pain and punished for reporting higher pain compared to their pretest pain levels. The control groups did not receive any consequences. Our results revealed placebo hypoalgesia was induced by operant conditioning only in the experimental groups with social and token-based reinforcement, compared to the control groups. The hypoalgesic effect found in the group that received verbal reinforcement did not differ significantly from the control group with the placebo application. Moreover, expectations about upcoming pain intensity were found to be a mediator, and the number of reinforcers received during conditioning was a predictor of placebo hypoalgesia. These findings highlight the potential benefits of incorporating token-based and social consequences for optimizing treatment outcomes in pain management.

## Introduction

There is a growing body of research that supports the role of learning processes in placebo effects in pain. Placebo hypoalgesia has been induced by classical conditioning (^1,2^; for a review, see:^3^), social observational learning (^4,5^; for a review, see:^6^), and verbal suggestions (^7,8^; for a review, see:^9^). Recently, another basic learning process – operant conditioning – has been shown to be its mechanism (^10^; for a review, see:^11^), as well as of hyperalgesia or allodynia (^12^; for review, see:^13^) and hypoalgesia^14^.

In operant conditioning, behavior is shaped through its consequences: a behavior that is followed by a reward (e.g., social approval) is more likely to occur in the future, whereas a behavior followed by a punisher (e.g., a reprimand) is less likely to occur in the future^15^. Placebo effects can also be shaped through the consequences of one’s pain behavior^11^. In a study by Adamczyk and colleagues^10^, participants were rewarded for reporting lower pain and punished for reporting higher pain following a placebo, which resulted in placebo hypoalgesia.

In the real world, both patients and healthy individuals encounter an unlimited number of possible rewards and punishers for their behavior, such as food, social attention, money, and the opportunity to engage in preferred activities^16^. The only existing study on placebo hypoalgesia induced by operant conditioning^10^ employed verbal feedback (e.g., 'Good!' or 'Bad!' displayed on the screen) as a form of reinforcement. It has been also demonstrated in other research fields that praise can effectively increase the frequency of desired behaviors in individuals^17^.

However, individuals dealing with pain-related issues are exposed to other reinforcers than just verbal feedback. Fordyce identified three potential rewards for chronic pain behavior: attention from others, relief from pain, and financial gain^18^. Social signs of approval and disapproval (conveyed through eye contact, physical contact, or facial expressions) have been proven to be effective in shaping behavior^15^, but the existing studies were carried out on very small samples (e.g.,^19^). The distribution of tokens, akin to financial gain, is a common strategy for reinforcing behavior change in different environments (psychiatric wards, classrooms^20^). Unfortunately, research that aims to compare the effectiveness of these different types of reinforcements is scarce, and in the context of pain and placebo research, it is non-existent. For that reason, we have chosen to investigate the effectiveness of the reinforcers employed in the previous study^10^ as well as two other types that are considered pivotal in a patient's life^18^: social and token-based reinforcement, in an experimental setting.

We hypothesized that social and token-based reinforcers would induce stronger placebo hypoalgesia than verbal reinforcers as social punishers have been shown to be more effective in influencing behavior than verbal reprimands alone^21^, and token-based reinforcement is believed to additionally increase one's intrinsic motivation^22^. Moreover, considering the existing evidence that expectations play a role in operant conditioning^23^ and that placebo effects are mediated by expectancies^24^, we hypothesized that expectations mediate placebo hypoalgesia induced by operant conditioning. We also hypothesized that placebo hypoalgesia would not extinguish over time and that the number of received reinforcers would predict the magnitude of the effect.

## Results

### Descriptive statistics

The analysis of descriptive statistics showed that the groups did not differ in BMI (*F*(4, 144) = 1.44, *p* = 0.23) or the level of pain during the pretest phase (*F*(4, 144) = 0.70, *p* = 0.59). Although a significant effect was found for age (*F*(4, 144) = 2.61, *p* = 0.038), the post-hoc comparisons between groups with Bonferroni correction applied showed that there were no significant differences between the groups in terms of the age of participants. No differences were found between groups in terms of sex (χ^2^ = 0.55, *p* = 0.97), education (χ^2^ = 16.12, *p* = 0.45), or job situation (χ^2^ = 8.56, *p* = 0.38).

### Placebo hypoalgesia induction

**Figure 1:**
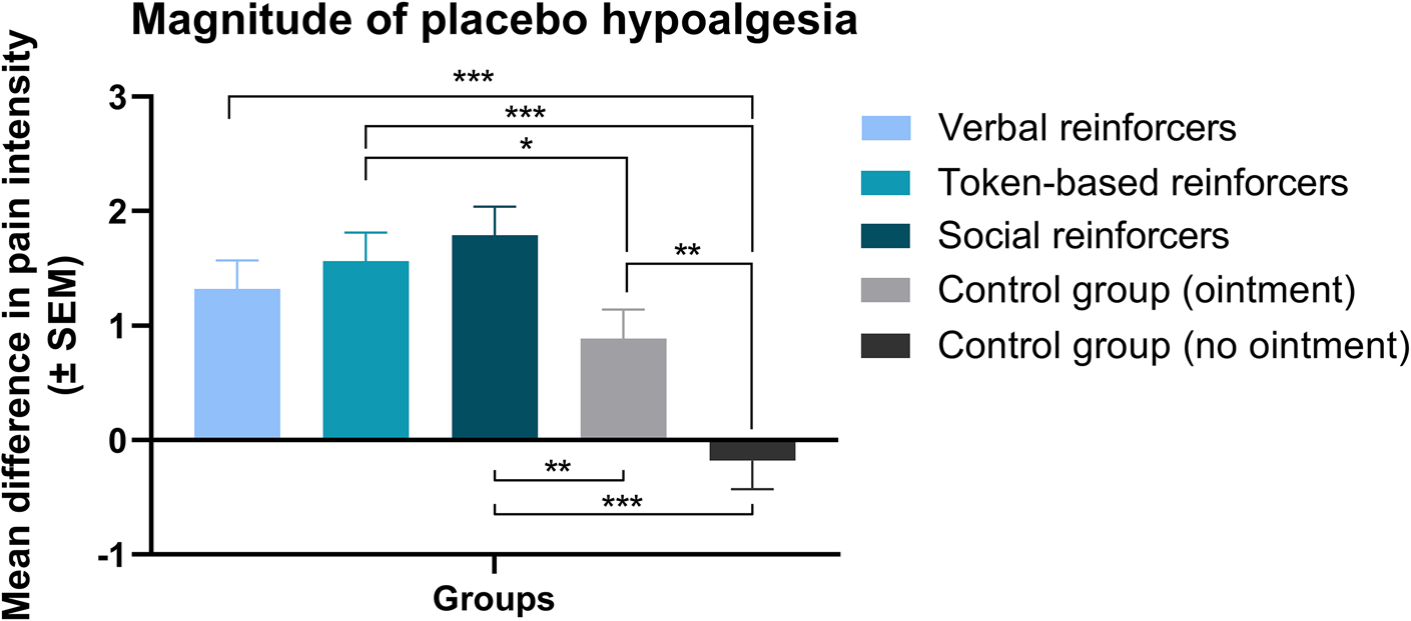
The mean difference in pain intensity ratings on the NRS between the pretest and the posttest in each of the study groups. A statistically significant difference in pain intensity change from the pretest to the posttest was observed between the following groups: all the experimental groups (verbal, token-based, and social) and the control group without the ointment, and between both the token-based and social group and the control group with the ointment (*** *p* < 0.001; **p < 0.01; *p < 0.05). Thus, placebo hypoalgesia was found to be induced by operant conditioning only in the groups with the token-based and social reinforcers.

**Figure 2:**
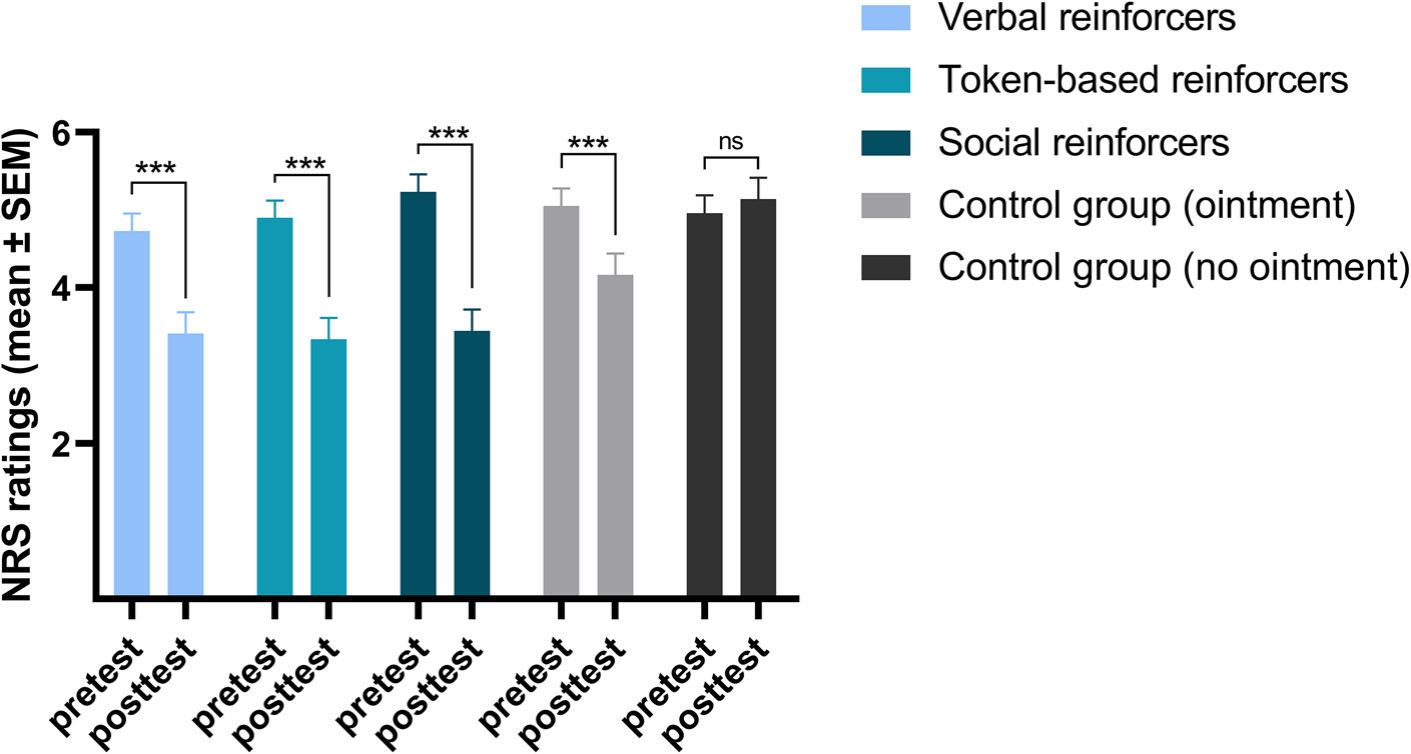
Mean pain intensity ratings on the NRS in the pretest and the posttest phases in groups. Participants in all the experimental groups, as well as the control group with the ointment, reported significantly lower pain intensity in the posttest compared to the pretest. However, no such effect was observed in the control group without the ointment (ns non-significant; *** *p* < 0.001).

To detect if placebo hypoalgesia was successfully induced through a repeated-measures ANOVA, we initially assessed the assumptions of normality, homogeneity of variance, and the presence of outliers. The homogeneity of variance assumption was satisfied, and no outliers were identified. While the normality assumption was partially violated, it is worth noting that ANOVA is known for its robustness against violations of this assumption^25^. A repeated-measures ANOVA on the pretest vs. posttest pain ratings revealed a statistically significant effect of ‘group’ (*F*(4, 144) = 3.33, *p* < 0.05, $\eta_{p}^{2}$ = 0.09), ‘phase‘ (*F*(1, 144) = 99.99, *p* < 0.001, *η*^*2*^_*p*_ = 0.41) and the interaction between the ‘phase’ and ‘group’ factors (*F*(4, 144) = 10.28, *p* < 0.001, $\eta_{p}^{2}$ = 0.22). The planned comparisons showed that the difference in pain ratings was significant between the following groups: (1) with verbal rewards and punishers and the control group without the ointment (*F*(1, 144) = 19.18, *p* < 0.001, $\eta_{p}^{2}$ = 0.12); (2) with token-based rewards and punishers and the control group without the ointment (*F*(1, 144) = 25.92, *p* < 0.001, $\eta_{p}^{2}$ = 0.15); (3) with token-based rewards and punishers and the control group with the ointment (*F*(1, 144) = 3.94, *p* < 0.05, $\eta_{p}^{2}$ = 0.03); (4) with social rewards and punishers and the control group without the ointment (*F*(1, 144) = 33.18; *p* < 0.001, $\eta_{p}^{2}$ = 0.19); (5) with social rewards and punishers and the control group with the ointment (*F*(1, 144) = 7.07, *p* < 0.01, $\eta_{p}^{2}$ = 0.05). The difference between the control group without the ointment and the control group with the ointment was found to be significant (*F*(1, 144) = 9.76, *p* < 0.003, $\eta_{p}^{2}$ = 0.06). The difference between the group with verbal rewards and punishers and the control group with the ointment was found to be insignificant (*F*(1, 144) = 1.61, *p* = 0.21, $\eta_{p}^{2}$ = 0.06). Therefore, the findings suggest that placebo hypoalgesia induced by operant conditioning was only observed in the experimental groups receiving token-based reinforcement, as well as the one receiving social reinforcement. In contrast, the hypoalgesic effect observed in the experimental group receiving verbal reinforcement did not differ significantly from that observed in the control group with the ointment. Consequently, it can be concluded that placebo hypoalgesia was not induced by operant conditioning in the experimental group receiving verbal rewards and punishers. Between-groups differences are depicted in Fig. 1, within-groups comparisons are depicted in Fig. 2, and detailed information is included in Table 1.

**Table 1:** One-way repeated measures ANOVA statistics of within and within-between planned comparisons.

		M	SD	*F*	df	*p*	eta^2^
Verbal R/P group	Pretest	4.73	1.08	30.24	1.144	< 0.001*	0.17
	Posttest	3.41	1.39				
Token-based R/P group	Pretest	4.90	1.17	42.42	1.144	< 0.001*	0.23
	Posttest	3.34	1.15				
Social R/P group	Pretest	5.24	1.35	55.76	1.144	< 0.001*	0.28
	Posttest	3.45	1.61				
Control with ointment group	Pretest	5.05	1.41	13.74	1.144	< 0.001*	0.09
	Posttest	4.17	1.66				
Control without ointment group	Pretest	4.96	1.08	0.54	1.144	0.46	0.004
	Posttest	5.14	1.56				
				*F*	df	*p*	eta^2^
Verbal r/p group versus token-based r/p group				0.51	1.144	0.47	0.004
Verbal R/P group versus social R/P group				1.94	1.144	0.17	0.01
Verbal R/P group versus control with ointment group				1.61	1.144	0.21	0.01
Verbal R/P group versus control without ointment group				19.18	1.144	< 0.001*	0.01
Token-based R/P group versus social R/P group				0.46	1.144	0.50	0.003
Token-based R/P group versus control with ointment group				3.94	1.144	< 0.05*	0.03
Token-based R/P group versus control without ointment group				25.92	1.144	< 0.001*	0.15
Social R/P group versus control with ointment group				7.07	1.144	< 0.01*	0.05
Social R/P group versus control without ointment group				28.57	1.144	< 0.001*	0.19
Control with ointment group versus control without ointment group				8.40	1.144	< 0.01*	0.06

R/P stands for Rewards/Punishers.

### Expectations as a mediator of placebo hypoalgesia induced by operant conditioning

**Figure 3:**
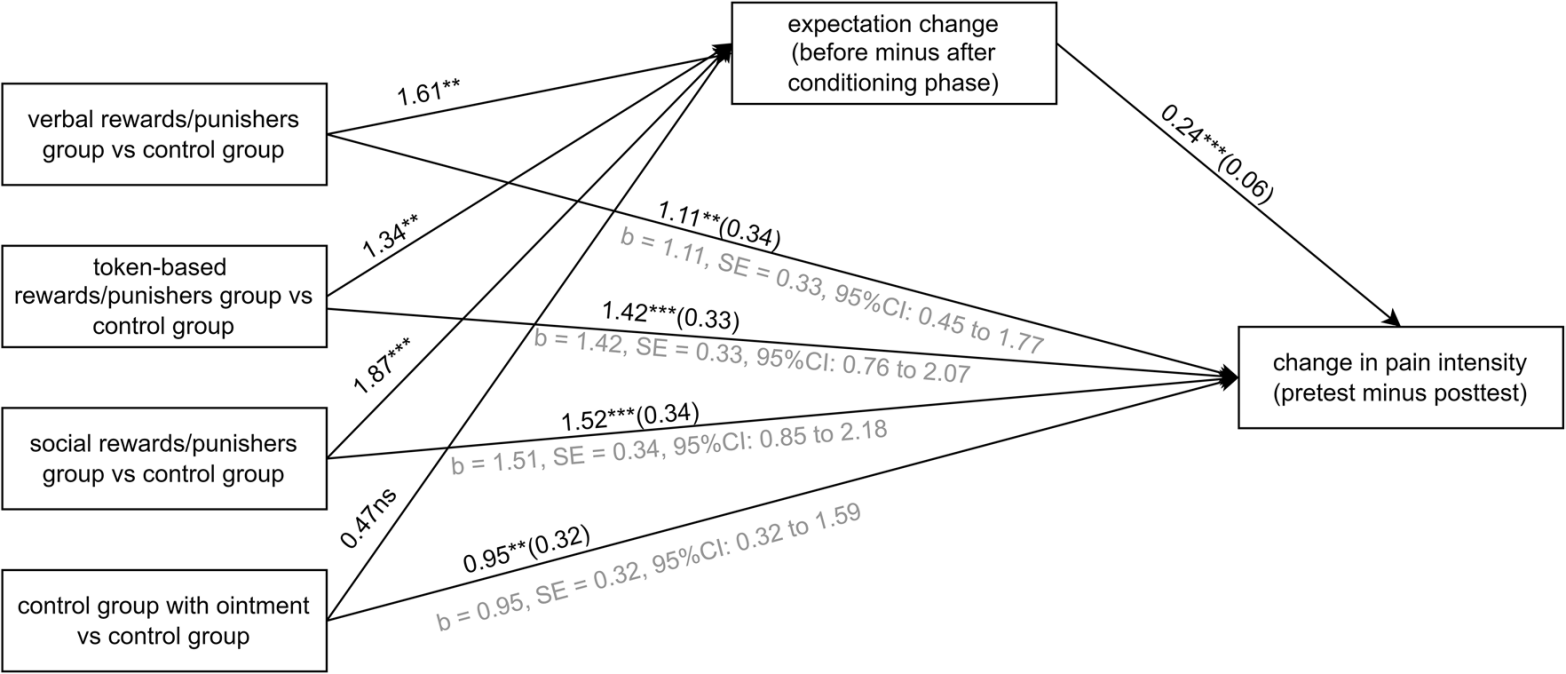
Mediation analysis of expectations in placebo hypoalgesia involving the groups (independent variable), expectation change (mediator), and change in pain intensity (dependent variable). Path values are path coefficients (standard errors). Placebo hypoalgesia was found to be partly mediated by the change in pain intensity expectation (ns non-significant; ***p < 0.001; **p < 0.01; *p < 0.05).

In the mediation analysis, the independent variable was coded as multicategorical with four categories (one for the control group with the ointment, and one for each of the experimental groups: with verbal, token-based, and social rewards and punishers) and with the control group without the ointment as a reference group. The results of the mediation analysis revealed a significant indirect effect of the group on the change in pain intensity (from the pretest to the posttest) through the change in pain intensity expectations only in the experimental groups (verbal: b = 0.388, SE = 0.16, 95% CI: 0.12 to 0.72; token-based: b = 0.324, SE = 0.15, 95% CI: 0.07 to 0.66; social: b = 0.453, SE = 0.17, 95% CI: 0.16 to 0.82). In the control group, the indirect effect was insignificant (b = 0.114, SE = 0.15, 95% CI: − 0.13 to 0.46). Furthermore, the direct effect of the group on the magnitude of the difference in pain intensity was also found to be significant in all the experimental groups (verbal: b = 1.109, *p* < 0.01; token-based: b = 1.416, *p* < 0.0001; social: b = 1.516, *p* < 0.0001). Thus, it can be concluded that the change in pain intensity from the pretest to the posttest in the experimental groups was partly mediated by the change in pain expectations (from the measurement before conditioning to the one after conditioning). The results are presented in Fig. 3.

### Extinction of placebo hypoalgesia

**Figure 4:**
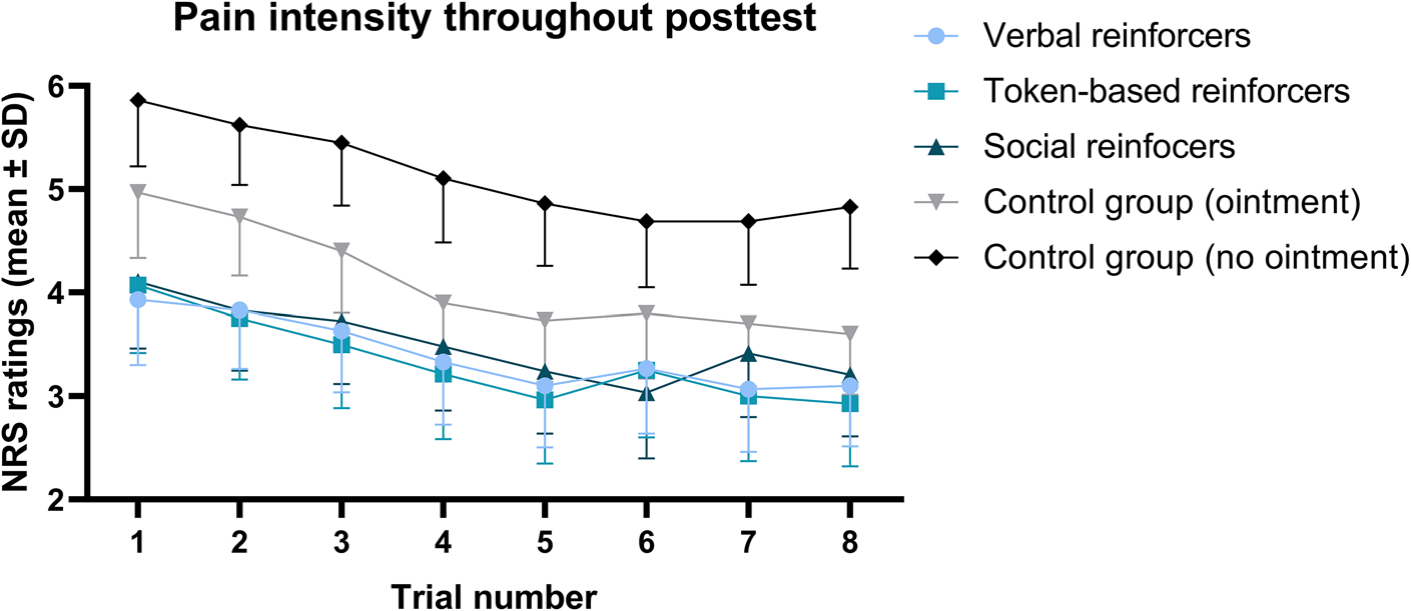
Pain intensity throughout the posttest phase in the experimental groups. No extinction of placebo hypoalgesia was found.

ANOVA for repeated measures on trial-by-trial pain ratings from the posttest phase in all the groups showed that placebo hypoalgesia was not extinguished in the experimental groups. In contrast, trials in the experimental groups (with verbal, token-based, and social reinforcers) were rated as less painful at the end of the posttest than trials at the beginning of that phase. However, in the control groups a similar pattern was observed as trials at the end of the posttest were rated as significantly less painful than at the beginning of the posttest. All the differences between trials within groups showed that pain intensity decreased during the posttest (see Fig. 4); these are presented in the supplementary materials (Supplementary Table 1). The main effects of group (*F*(4, 141) = 7.29, *p* < 0.001, $\eta_{p}^{2}$ = 0.17) and trial (*F*(7, 987) = 42.14,* p* < 0.001, $\eta_{p}^{2}$ = 0.23) were found to be significant; however, the interaction between group and trial was nonsignificant (*F*(14, 588) = 0.776, *p* = 0.70).

### Number of rewards as a predictor of the magnitude of placebo hypoalgesia

The forward stepwise regression analysis was performed in all the experimental groups combined, with the number of received rewards as an independent variable and the difference between the mean pain intensity in the pretest and the posttest as a dependent variable. It revealed that the number of rewards was a significant predictor of placebo hypoalgesia (β* = 0.669; corr. R^2^ = 0.44, *F*(1, 87) = 70.38, *p* < 0.001). Furthermore, to verify whether the number of rewards was a significant predictor of the difference in pain intensity between the pretest and the posttest in the experimental groups in which operant conditioning significantly contributed to placebo hypoalgesia, the regression analysis was conducted separately in those groups. The results showed that the number of rewards received during the conditioning phase was a significant predictor of the change in pain intensity from the pretest to the posttest in the token-based reinforcers group (β* = 0.703, corr.R^2^ = 0.48, *F*(1, 28) = 27.40, *p* < 0.001) and in the social reinforcers group ( β* = 0.717, corr.R^2^ = 0.50, *F*(1, 27) = 28.49, *p* < 0.001).

### Personality traits

The correlation analyses revealed that only one subscale of the TIPI scale (Conscientiousness) significantly and negatively correlated with the magnitude of placebo hypoalgesia (r =  − 0.23, *p* < 0.003). The results of the other TIPI subscales, as well as SPRRS and TAS, were not significantly correlated with the magnitude of the effect and are reported in the supplementary materials together with the Cronbach’s α levels obtained in our study (see Supplementary Table 2).

### End survey analysis

The analysis of the post-study questionnaire (refer to Table 2) revealed that only 4.03% of participants figured out the real aim of the study. What is more, 35% believed that the ointment had an impact on their pain sensation (Q1), with 76.19% reporting that it caused pain relief and 23.81% indicating an increase in pain (Q2). The majority of participants (78.33%) stated that the additional device attached to their forearm measured the pain intensity correctly (Q3). Subsequent analyses investigating the potential confounding effects of participants’ beliefs regarding the ointment’s influence (examined through ‘group’ x ‘phase’ x ‘Q1’ interaction (*F*(3, 112) = 0.688, *p* = 0.56) and ‘group’ x ‘phase’ x ‘Q2’ interaction *(F*(3,34) = 0.26, *p* = 0.86)), and their beliefs about the device's pain assessment accuracy (*F*(3, 112) = 0.28, *p* = 0.84) revealed that participants’ beliefs did not confound the obtained results.

**Table 2:** Results of the end of study questionnaire.

Question at the end of the survey		Verbal group	Token-based group	Social group	Control group with ointment	Control group without ointment	Total
Participants who figured the aim of the study		3.33% (1)	10.00% (3)	6.67% (2)	0% (0)	0% (0)	4.03% (6)
Q1: Did the ointment influence your pain sensation?	No	70.00% (21)	70.00% (21)	56.67% (17)	63.33% (19)	–	65.00% (78)
	Yes	30.00% (9)	30.00% (9)	43.33% (13)	36.67% (11)	–	35.00% (42)
Q2: How did the ointment influence your pain sensation?	Relieved pain	23.33% (7)	20.00% (6)	33.33% (10)	30.00% (9)	–	76.19% (32)
	Increased pain	6.67% (2)	10.00% (3)	10.00% (3)	6.67% (2)	–	23.81% (10)
Q3: Did the device correctly measure your pain sensation intensity?	No	20.00% (6)	26.67% (8)	30.00% (9)	10.00% (3)	–	21.67% (26)
	Yes	80.00% (24)	73.33% (22)	70.00% (21)	90.00% (27)	–	78.33% (94)

Participants responded to Q2 only when their answer to Q1 was ‘Yes’.

## Discussion

The present study tested the effect of different types of rewards and punishers on placebo hypoalgesia induced by operant conditioning. In the operant conditioning paradigm, the likelihood of a behavior (in this case a certain reaction to pain) increases when followed by a reward and diminishes when followed by a punisher. Thus, a voluntary behavior is being reinforced or punished, in contrast to classical conditioning where an automatic, involuntary association is created through repeated pairing of stimuli.

In our study, we conducted a comparison of three types of rewards and punishers: verbal, social, and token-based. We specifically selected verbal reinforcers for replication purposes, as they were used in the only existing study on placebo hypoalgesia induced by operant conditioning^10^. Since another objective was to investigate whether more ecologically valid reinforcers could induce placebo hypoalgesia in an experimental setting, we opted to compare verbal reinforcers with social and token-based ones. This choice was made because token-based and social reinforcers partially simulate reinforcement in the form of money and attention from others, which are known to be crucial factors in shaping the behavior of chronic pain patients^18^.

We found that rewards and punishers such as social cues and tokens can induce placebo hypoalgesia in the operant conditioning paradigm. In contrast, the hypoalgesic effect induced in the group receiving verbal rewards and punishers did not differ significantly from the control group that received ointment application, suggesting that these reinforcers did not significantly contribute to the induction of placebo hypoalgesia. Moreover, to the best of our knowledge, this is the second study to date in which placebo hypoalgesia has been induced by operant conditioning.

Social reinforcers (signs of approval and disapproval) are generalized conditioned reinforcers that are most commonly used in applied behavior analysis^26^. They are usually conveyed through eye contact, physical contact, and facial expressions, all of which wield powerful influence in shaping desired behavior^15^. Social rewards (conveyed to participants in the form of a video clip similar to the one used in our study) were shown to cause an improvement in motor skill memory that had direct effects on the consolidation process^27^. What is more, verbal reprimands were found to be more effective in suppressing behavior when combined with eye contact and physical contact than verbal reprimands alone^21^. While our study focused on the separate use of different types of reinforcement, one may notice a tentative correspondence with our results, as social reinforcers contained non-verbal cues about the correctness of pain behavior, effectively conveying a similar message to the verbal ones. Also, Kazdin and Klock^28^ found that social rewards (smiling and physical contact) enhanced the effectiveness of verbal approval in shaping classroom behavior.

There are a few reasons why social rewards and punishers might be more powerful in influencing behavior than verbal ones. Firstly, they convey some additional information, namely an emotional component, in addition to the mere feedback received as a result of one’s actions, as is the case for verbal rewards and punishers. The approval gained from others can lead to increased self-efficacy^29^ and motivation^27^. On the other hand, disapproval can result in feelings of shame, guilt, and decreased self-worth and a disapproving facial expression indicates a negative evaluation that signals to a person that she or he has done something socially undesirable, thus leading individuals to change their behavior to avoid further criticism^30^. Therefore, it seems plausible that the approval and disapproval expressed by the experimenter during the conditioning process enhances the effectiveness of conditioning through its emotional and motivational factors.

Another type of rewards and punishers that in our study appeared to be effective in inducing placebo hypoalgesia was based on a token economy. Gaining tokens acts as positive reinforcement, increasing the likelihood that the desired behavior will be repeated, whereas losing tokens acts as negative punishment, thus decreasing this likelihood. A token economy can be particularly effective in situations where the reward should be immediate, as has been proven to work in environments such as psychiatric hospitals or correctional facilities^20^. Similar to social reinforcement, token-based reinforcement not only provides feedback about one’s performance but can also involve a motivational factor by increasing one’s intrinsic motivation^22^. Another study^31^ showed that the simple accumulation of recorded points used as rewards was almost as effective in shaping stuttering behavior as rewards that could be exchanged for tangible rewards (toys or verbal praise). In our study, even though the participants could not exchange the tokens they gained for anything tangible, the act of receiving these virtual tokens also served as a powerful reinforcement as it induced placebo hypoalgesia, in contrast to verbal reinforcement. Also, the design of the procedure allowed the participants to always keep track of the total number of tokens they had received, which could have made the correspondence between their behavior and the consequence easier to notice.

However, a previous study on placebo hypoalgesia induced by operant conditioning^10^ reported a significant effect of verbal reinforcement, which differs from our findings. It’s essential to note that the verbal reinforcement in our study involved only one-word prompts displayed on the screen, either ‘Good!’ as a reward or ‘Bad!’ as a punisher. In contrast, the verbal reinforcement used in the prior study conveyed additional information (‘Too high!’/’Too low!’), which we could not implement in our study due to the inclusion of nonverbal reinforcement in other groups and differences in study design. Additionally, the placebo used in the previous study took an abstract, non-medically connoted form, namely, a color displayed on the screen, whereas we employed a medically connoted placebo (ointment). There is evidence suggesting that the type of placebo may influence the magnitude of placebo hypoalgesia as studies using medically-connoted placebos tend to show larger effects than those employing non-medically connoted ones^32^. This could also be the cause of the difference in results observed in the two studies. Consequently, future research could explore this issue further by comparing different types of placebos in inducing placebo hypoalgesia within the operant paradigm. Additionally, investigating various types of verbal reinforcers could provide valuable insights, as well as the effect of different types of reinforcers combined.

In the first study that examined placebo hypoalgesia induced by operant conditioning^10^, an extinction of the effect was not found as there was no change in its magnitude over time. We confirmed this result, as pain intensity throughout the posttest did not increase but actually diminished in the experimental groups. On the other hand, the pattern in the control groups was similar, with pain being rated as significantly lower at the end of that phase than at the beginning. One explanation would be that this result was due to a habituation process. On the other hand, the difference in pain intensity between the pretest and the posttest was statistically insignificant only in the control group without the ointment. In contrast, all other groups showed a significant reduction in pain intensity from the pretest to the posttest, as illustrated in Fig. 2. These results suggest that the habituation process is less likely to be solely responsible for the decrease in pain intensity during the posttest. What is more, in studies on the placebo effect induced by classical conditioning, the effect has been proven to be more resistant to extinction when a partial rather than a continuous schedule of unconditioned stimuli was used ^33^. Thus, future research should attempt to determine whether the non-continuous schedule of reinforcement (intermittent) could indeed induce even more robust and resilient placebo hypoalgesia by operant conditioning.

Expectations regarding pain relief may be derived from information acquired, e.g., through learning processes, and are believed to mediate placebo effects^34^ and lead to the formation of placebo response^35^. Several studies have shown that expectations about upcoming pain contributed to placebo effects induced by classical conditioning (for review, see:^3^) and mediated placebo hypoalgesia^36,37^. Moreover, there is evidence indicating that expectations are involved in operant learning^23^. Thus, we hypothesized that a similar relation would occur in the case of operant conditioning of placebo hypoalgesia. Indeed, we found that a change in expectations regarding the upcoming pain before versus after the conditioning phase partly mediated placebo hypoalgesia in the experimental groups. Interestingly, while there was no significant difference in pain change from the pretest to the posttest between the verbal reinforcement group and the control group with the ointment, the change in expectations served as a significant mediator only within the verbal reinforcement group and not within the control group with the ointment. This suggests that our manipulation did induce expectations regarding upcoming pain intensity in the verbal reinforcement group, though they may not have been sufficient to contribute to placebo hypoalgesia. To sum up, this is the first study that found mediation of expectations in placebo hypoalgesia induced by operant conditioning; secondly, it adds to the existing body of knowledge on the role of expectations in placebo hypoalgesia acquisition through learning processes.

Another hypothesis that we confirmed was that the number of rewards received during conditioning would be a significant predictor of the magnitude of placebo hypoalgesia. The bigger the number of rewards (and the smaller the number of punishers) received, the better the learning outcomes and the greater the magnitude of placebo hypoalgesia. Unfortunately, our study design does not make it possible to compare the efficacy of rewards and punishers separately because participants always received either a reward or a punisher; hence, the more rewards they received, the fewer punishers were administered to them. Future research should focus on separating the effects of these two types of consequences on the magnitude of placebo hypoalgesia induced by operant conditioning.

Another interesting result that appeared in our study was the occurrence of a hypoalgesic effect as a result of the mere application of the placebo ointment that did not contain any analgesic properties. Furthermore, participants did not receive any information regarding the properties of the ointment. Yet, we found a significant difference in pain ratings between the control groups: participants in the group in which the ointment was applied felt less pain than participants in the group in which no ointment was applied. Thus, it can be concluded that the mere application of the ointment by the experimenter induced placebo hypoalgesia. This result is in line with research showing that contextual factors, such as a therapeutic ritual around a patient’s treatment, influence brain activity and the therapeutic outcome^39^. Unfortunately, we did not measure participants’ expectations of pain intensity right after the ointment was applied, thus we may only hypothesize that previous treatment experiences induced expectations of pain alleviation after the ointment application as they are believed to be central elements to placebo hypoalgesia^38^. This finding raises an important issue for placebo research, emphasizing the necessity of including multiple control groups in studies in order to distinguish between placebo effects acquired through the experimental manipulation from the contextual effects, especially when medically connoted agents are used as placebos.

Furthermore, we observed a negative correlation between conscientiousness and the magnitude of placebo hypoalgesia induced through operant conditioning. These findings are consistent with the results obtained by Beedie, Foad, and Coleman^40^, who reported negative correlations between both conscientiousness and agreeableness with sport performance in the placebo condition. However, it is worth noting that many previous studies investigating the placebo effect have not reported such a relationship^41^. Consequently, the current evidence remains inconclusive, underscoring the need for further research exploring the role of personality traits in placebo effects.

Some limitations of the study should be acknowledged. Firstly, the study was conducted in a laboratory setting and the generalization of its results to the clinical population should be done with caution. This basic science study represents an initial step in understanding the role of reinforcement in placebo hypoalgesia, and its findings should be further confirmed in research settings that are more ecologically valid. Secondly, only subjective measures of pain intensity and expectations (relying on self-reports) were examined in this study. Although according to the definition of pain^42^ (p. 1977), “pain is always a personal experience” and its measurement relies mostly on self-reports, the use of more objective measures such as biological markers in future studies would enrich the obtained data. Thirdly, the sample size was calculated for the needs of the main analysis only (the within-between interaction), thus it might have been insufficient to detect correlation effects or to fully estimate the mediation effect. Moreover, it is possible to speculate that the observed effect in the experimental groups could be attributed to response bias, where participants intentionally rated pain as less intense in order to receive reinforcement, without experiencing any actual changes in pain sensation. However, the data obtained during the posttest phase contradicts this hypothesis. Participants in the experimental groups consistently rated pain as less intense compared to their pretest ratings, even after the reinforcement process was discontinued. Furthermore, the responses to control questions provided by participants suggest a low likelihood of response bias, as only approximately 4% (6 participants) correctly deduced the true purpose of the study. Additionally, the majority of participants (78%) indicated that the device used to measure their “objective” pain intensity was accurate, as shown in Table 2.

In conclusion, the present findings show the importance of operant conditioning in inducing placebo hypoalgesia. To the best of our knowledge, our study is the second to confirm that patients can learn placebo effects as a result of operant learning. We showed that the investigated rewards and punishers (social approval, tokens) are powerful in changing pain experience through operant conditioning Those results may be clinically significant, as these are types of consequences that patients with chronic pain may encounter^18^. More research is needed to further examine operant conditioning as a mechanism of placebo effects in pain, as well as to test the variables that could enhance this effect.

## Materials and methods

The study protocol was approved by the ethics committee at the Institute of Psychology, Jagiellonian University, Cracow, Poland (decision no. KE/5_2022). All methods were performed in accordance with the relevant guidelines and regulations.

### Sample size

The sample size was determined based on the within-between interaction effect size (f = 0.20) derived from a previous study that investigated placebo hypoalgesia induced through operant conditioning ^10^. To detect a significant difference in pain intensity between the groups, it was estimated that a minimum sample of 30 participants was required per group (α = 0.05, 80%, corr. = 0.5, within-between interaction). The calculation was performed using G*Power 3.1.9.2 software^43^.

### Participants

A total of 161 healthy participants aged from 18 to 50 who were recruited through advertisements on social media took part in the study. The exclusion criteria were previous involvement in a pain experiment; studying or graduating psychology; the presence of pain on the day of the experiment; alcohol or drug abuse; having unremovable metal objects in the body; diagnosed neurological, cardiovascular, metabolic, musculoskeletal, or psychiatric disorders in the preceding six months; pregnancy. All criteria were evaluated through an online questionnaire that was distributed to participants at the recruitment stage. The participants were randomly assigned to five groups: three experimental and two control. We stopped the data collection after obtaining full data records from 30 participants in each group. Due to procedure and device errors that occurred during the testing of 12 participants, the total number of participants (161) is higher than the number of data records analyzed in this study (149).

Participants were randomly assigned to groups before their arrival at the laboratory, and the randomization process was conducted using an online random distributor. The experiment took place in the laboratory of Pain Research Group at the Institute of Psychology, Jagiellonian University in Kraków. Participants were informed that the purpose of the study was to assess their ability to rate subjective pain intensity in comparison to the objective level of pain intensity of the pain stimuli. Every participant provided written informed consent and was financially compensated for the participation in the experiment (30 PLN). The basic descriptive statistics of the study sample are shown in Table 3.

**Table 3:** Basic descriptive statistics of the study participants (mean ± standard deviation) or percentage (number) in the total sample and the groups separately.

Variables	Total Sample (*N* = 149)	Verbal R/P group (*N* = 30)	Token-based R/P group (*N* = 30)	Social R/P group (N = 30)	Control group with ointment (N = 30)	Control group without ointment (N = 30)
Age (years)	24.21 ± 5.23	22.47 ± 2.19	25.30 ± 6.34	23.90 ± 5.89	23.23 ± 2.86	26.21 ± 6.64
BMI (kg/m^2^)	22.63 ± 3.38	22.00 ± 3.96	23.48 ± 3.58	22.48 ± 3.06	21.89 ± 2.41	23.32 ± 3.59
Sex						
Male	40.67% (61)	36.67% (11)	43.33% (13)	43.33% (13)	43.33% (13)	36.67% (11)
Female	59.33% (89)	63.33% (19)	56.67% (17)	56.67% (17)	56.67% (17)	63.33% (19)
Education						
Primary school	2.01% (3)	0% (0)	6.67% (2)	0% (0)	0% (0)	3.45% (1)
Secondary school	51.01% (76)	56.67% (7)	43.33% (13)	43.33% (13)	63.33% (19)	48.28% (14)
Vocational school	3.36% (5)	3.33% (1)	0% (0)	10.00% (3)	0% (0)	3.45% (1)
Post-secondary school	16.11% (24)	20.00% (6)	16.67% (5)	20.00% (6)	13.33% (4)	10.34% (3)
Higher education	27.52% (41)	20.00% (6)	33.33% (10)	26.67% (8)	23.33% (7)	34.48% (10)
Job situation						
Student	69.80% (104)	80.00% (24)	63.33% (19)	70.00% (21)	80.00% (24)	55.17% (16)
Employed	22.15% (33)	10.00% (3)	30.00% (9)	23.33% (7)	16.67% (5)	31.03% (9)
Unemployed	8.05% (12)	10.00% (3)	6.67% (2)	6.67% (2)	3.33% (1)	13.79 (4)

R/P stands for rewards/punishers.

For education, the highest obtained level was taken into account.

### Stimuli and measures

#### Pain stimuli

The thermal pain stimuli were delivered through a contact thermode by the Pathway Pain & Sensory Evaluation System, model ATS (Medoc Ltd Advanced Medical System, Israel) to the inner side of the nondominant forearm. Each pain stimulus lasted 3 s (in its plateau phase).

#### Placebo

Placebo was applied to participants’ forearm in the form of an inert ointment with a thyme scent. Participants were not provided with any information regarding the properties of the ointment, with the exception that it is safe for use on the skin.

#### Sham device

In order to justify the apparent aim of the study, we presented to participants a sham device that allegedly provides an objective measurement of pain and allows for the comparison of participants’ subjective pain ratings and the objective level of pain intensity. It consisted of two electrodes with cables that were attached to the same forearm as the thermode.

#### Rewards and punishers

**Figure 5:**
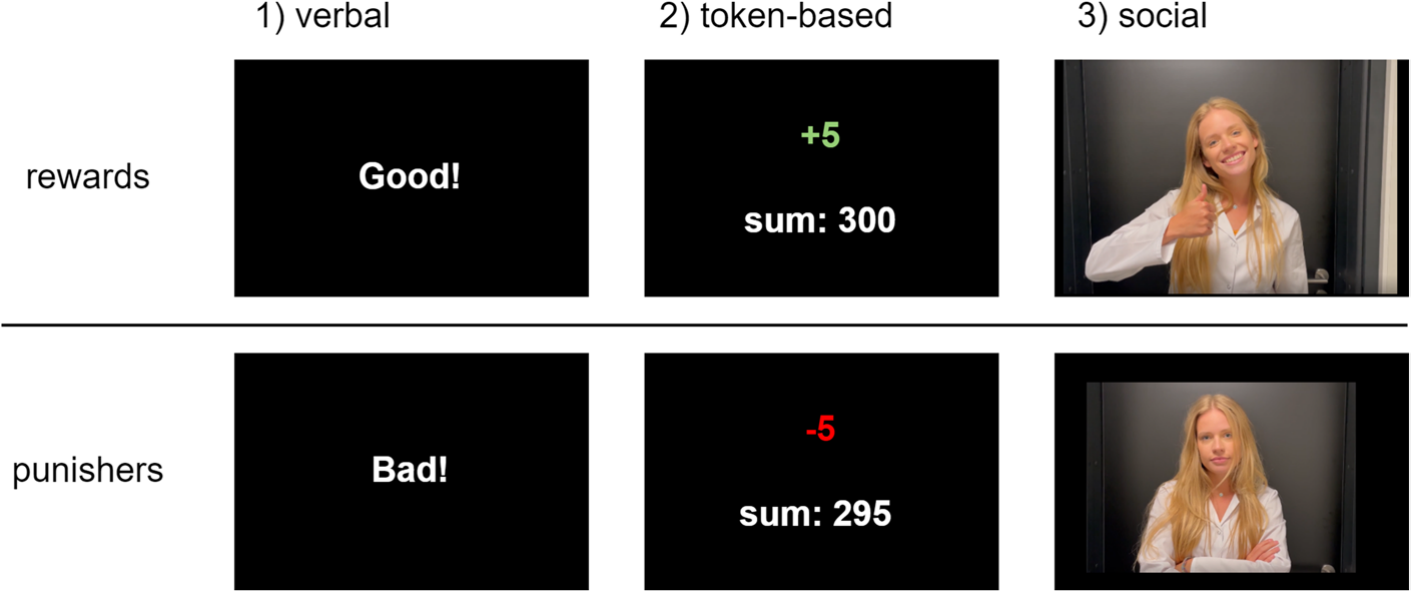
Types of reinforcers used in three experimental groups: (**A**) verbal, (**B**) token-based, and** C**) social.

During the operant conditioning phase, different types of rewards and punishers were presented on the screen (for the duration of 3 s) in the experimental groups. In the first experimental group, participants received verbal rewards and punishers in the form of words shown on the computer screen (“Good!”, “Bad!”). In the second experimental group, participants received token-based rewards and punishers: at the beginning of the procedure, they had 300 tokens on their account; this amount could increase (+ 5 as a reward) or decrease (− 5 as a punisher). The account balance was visible throughout the duration of the whole manipulation phase. In the third experimental group, participants received social rewards and punishers in the form of short videos lasting 3 s that presented the experimenter nonverbally expressing a sign of approval (as a reward) or disapproval (as a punisher). We chose to display prerecorded video stimuli instead of performing a live reinforcement process to ensure standardized conditions for all participants. The simplified forms of the rewards and punishers displayed on the screen are shown in Fig. 5.

#### Pain ratings

Throughout the experiment, participants rated pain intensity (“How intense was the pain you felt?”) and pain expectation (“How intense pain stimulation do you expect?”) on an 11-point Numeric Rating Scale (NRS) ranging from 0 = ‘no pain’ to 10 = ‘the most intense tolerable pain’. All the ratings were provided verbally by participants.

#### Questionnaires

In order to explore personality variables that may potentially influence placebo hypoalgesia induced by operant conditioning, three questionnaire measures were applied in the study. The Sensitivity to Punishment and Sensitivity to Reward Questionnaire (SPSRQ)^44^ is a measurement of the behavioral approach system (BAS) and behavioral inhibition system (BIS) that consists of two scales (BAS and BIS) and 21 items. The respondents provide answers in the yes/no format. The Polish adaptation of the scale^45^ has been validated, and its reliability is satisfactory (Cronbach’s α for the BAS scale is 0.71; for the BIS scale it is 0.84). The minimum number of obtained points for both scales is 0; the maximum is 11 for the BAS scale and 13 points for the BIS scale. The Ten Item Personality Inventory^46^ (TIPI) is a short method used to measure the “Big Five” personality traits (neuroticism, extraversion, conscientiousness, openness to experience, and agreeableness). Respondents are asked to answer each of the self-report statements on a 7-point Likert scale (from 1 – Strongly Disagree to 7 – Strongly Agree). Cronbach-alphas of the Polish version of TIPI^47^ are: 0.44 for Openness to Experience, 0.58 for Agreeableness, 0.68 for Extraversion, 0.74 for Emotional Stability, and 0.75 for Conscientiousness; test–retest reliability ranged from 0.56 (Openness to Experience) to 0.83 (Emotional stability and Conscientiousness). In each scale, the number of points that are possible to obtain ranges from a minimum of 2 to a maximum of 14. The Tellegen Absorption Scale^48^ (TAS) measures imaginative involvement and the tendency to become mentally absorbed in everyday activities. This scale consists of 37 items, and respondents provide answers in the yes/no format, with a total of 37 possible points to obtain. The scale was translated into Polish by Jerzy Siuta.

### Design and procedures

**Figure 6:**
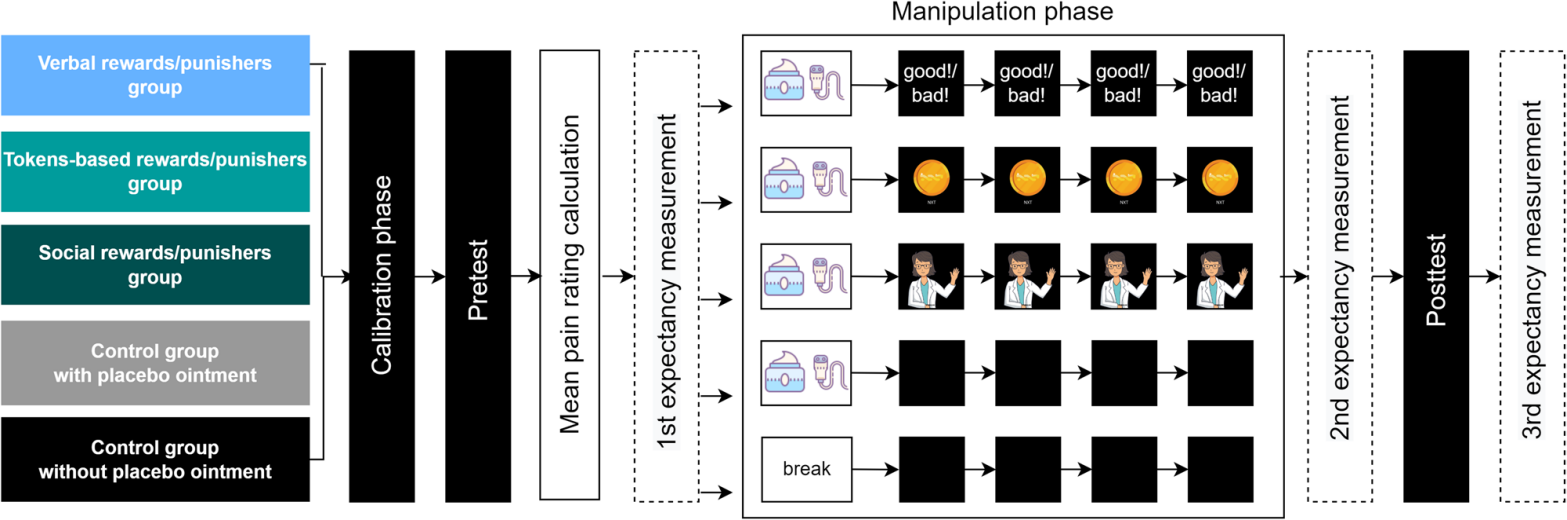
Study design. The study involved three experimental groups (with verbal, token-based, and social rewards and punishers) and two control groups (one without the ointment application, and one with the ointment application). Participants in the experimental groups underwent an operant conditioning phase in which different rewards and punishers were used during learning. Participants in the control groups did not undergo the operant conditioning phase.

The experiment consisted of five groups (three experimental and two control groups) and four phases: calibration, pretest, manipulation (operant conditioning), and testing. The experimental design is presented in Fig. 6. The procedure was programmed using Python 3 language and PsychoPy 2021 software^49^.

#### Calibration

During the calibration phase, one series of ascending and one series of descending thermal stimuli were distributed to participants. Each target temperature was kept constant for 3 s (plateau phase), preceded by a ramp-up increase and followed by a ramp-down decrease to baseline temperature (32 °C/s) at a rate of 10 °C/s. Participants rated each stimulus on the NRS. The starting point of the ascending series of stimuli was 40 °C; from there on, the stimuli were applied at 5-s intervals, increasing in temperature by half a degree Celsius degree each time until the maximum temperature was reached (50 degrees) or until the participant rated the intensity of the pain stimulus as 8 on the NRS. Following that, a descending series of pain stimuli was applied, starting from the temperature that was last applied in the first (ascending) series, until the participants rated pain intensity as 0 or 1 on the NRS, or when the temperature of 40 °C was reached. The data obtained from the calibration was used to estimate an intensity corresponding to 5 on the NRS by fitting the obtained pain intensity ratings to an exponential curve.

#### Pretest

During the pretest phase, 10 stimuli of the same intensity (corresponding to 5 on the NRS) were applied to the participants’ forearm. Each time, participants were asked to rate the pain intensity on the scale. At the end of this phase, pain intensity expectancy was measured. Also, the mean pain intensity rating was calculated and used as a reference point in the manipulation phase (in the experimental groups).

#### Manipulation phase

At the beginning of the manipulation phase, in the experimental groups and one control group, the placebo ointment was applied to the participants’ forearm (under the place where the thermode was attached) and the sham device was attached. Participants did not receive any information regarding the ointment. After a short break that allowed for the ointment to dry, the experimental groups underwent the operant conditioning procedure: they received a reward every time they rated the pain intensity as lower than in the pretest; they received a punisher every time they rated the pain intensity as higher or equal than in the pretest. The rewards and punishers differed between the experimental groups (see Fig. 1). In the first control group, the placebo ointment was applied and the sham device was attached to the participants’ forearm; however, participants did not receive any rewards or punishers during this phase. Participants were also not informed about the nature of the ointment or the electrode. In the second control group, no ointment was applied and the sham electrode was not attached, but the same amount of time passed between the pretest and the conditioning phase. In the control groups, each time after participants rated the pain intensity, a blank screen was displayed instead of a reward or punisher for 3 s. In all the groups, participants underwent 4 series of 8 pain stimuli each. At the end of this phase, the second expectancy measurement was carried out.

#### Posttest phase

The posttest phase was identical to the pretest phase, except that only 8 stimuli were delivered. At the end of this phase, pain expectancy was measured for the third time. Subsequently, participants were asked to fill in the SPSRQ, TIPI, and TAS questionnaires (only the experimental groups, see Materials and methods) and some control questions: (1) “What, in your opinion, was the aim of the study?”; (2) “Did the ointment influence your pain experience in any way?”; (3) (if the answer to question 2 was ‘yes’) “How did it influence your pain experience?”; and (4) “Did the objective pain intensity level device measure your pain level accurately”.

### Statistical analysis

Descriptive statistics were calculated for the following variables: age, body mass index (BMI) (means and standard deviations), education level, employment status, and the NRS ratings from the pretest; this was followed by analyses of differences between the groups using the one-way ANOVA with post hoc multiple comparisons for age, BMI, and the NRS ratings from the pretest and the chi-square test for education level and employment status. Bonferroni correction was applied to all the possible pairwise comparisons.

To detect if placebo hypoalgesia was successfully induced in the experimental groups, the pretest and the posttest pain ratings were compared through the repeated-measures ANOVA with ‘group’ (verbal, token-based, social, control with the ointment, control without the ointment) as a between-subject factor and ‘phase’ (pretest, posttest) as a within-subject factor. *F* tests were then followed by planned comparisons involving all the between-group and within-group comparisons.

To examine whether the effect of operant conditioning on pain ratings was mediated by expectancy, a mediation analysis with the groups as an independent variable, expectation change as a mediator, and change in pain intensity from the pretest to the posttest as a dependent variable was conducted using PROCESS Procedure for SPSS version^50^.

To detect the possible extinction of placebo hypoalgesia, a repeated-measures ANOVA was conducted in all the groups on pain intensity, with ‘group’ (verbal, token-based, social, control with the ointment, control without the ointment) as a between-subject factor and ’trial’ in the posttest (from 1 to 8) as a within-subject factor. Subsequent planned comparisons were conducted between all the variables.

To assess the influence of the number of rewards received during the operant conditioning procedure, we conducted a regression analysis with the number of rewards as a predictor, and the difference in pain intensity between the pretest and the posttest (in the experimental groups) as a dependent variable. The design of the study did not allow for a separate analysis of the number of rewards and punishers received (as the more rewards one received, the fewer punishers were administered to them), hence only the number of rewards was analyzed.

To investigate the effects of individual differences on the magnitude of placebo hypoalgesia induced by operant conditioning, a correlation analysis was performed in the experimental groups, with SPQRS, TIPI subscale scores, and TAS score as independent variables, and the difference between pretest and posttest as a dependent variable. Moreover, Cronbach’s α levels were calculated for the TIPI scores obtained in our study. To examine whether participants’ beliefs regarding the action of the ointment (Q1, Q2) and the perceived accuracy of the additional device allegedly measuring their objective pain level (Q3) could have confounded the results, three separate repeated-measures ANOVAs were performed with each of the questions (Q1, Q2, Q3) as an additional factor, ‘group’ as a between-subject factor and ‘phase’ (pretest, posttest) as a within-subject factor. The analyses were carried out in the STATISTICA data analysis software system, 64-bit, version 13 (StatSoft Inc., Tulsa, OK, USA) and IBM SPSS Statistics.

### Supplementary Information

Supplementary Information.

## Data Availability

The data set used in analyses is available at RODBUK Cracow Open Research Data Repository (https://doi.org/10.57903/UJ/ATW8NS).
