# Supplementary material for: Placebo hypoalgesia induced by operant conditioning: a comparative study on the effects of verbal, token-based, and social rewards and punishers
Source: Sci Rep. 2023 Nov 21;13:20346. doi: 10.1038/s41598-023-47482-1 (PMC10663593; doi:10.1038/s41598-023-47482-1)
Supplement: Supplementary file 1 — Supplementary Information. [file 41598_2023_47482_MOESM1_ESM.pdf]

Placebo Hypoalgesia Induced by Operant Conditioning: A Comparative Study on the Effects of Verbal, Token-Based, and Social Rewards and Punishers

Helena Bieniek[1,2*], Przemysław Bąbel[1]

[1] Jagiellonian University, Institute of Psychology, Pain Research Group, Kraków, Poland
[2] Jagiellonian University, Doctoral School in the Social Sciences, Kraków, Poland

Supplementary Table 1. Mean difference and SEM values in groups (verbal, token-based and social rewards and punishers, control group with ointment, control group without the ointment) within posttest trials (1–8). Statistically significant differences are marked with *.

Verbal reinforcers group					Token-based reinforcers group					Social reinforcers group					Control group with ointment					Control group without ointment				
Trial nr	Trial nr	Mean diff.	SEM	*p*	Trial nr	Trial nr	Mean diff.	SEM	*p*	Trial nr	Trial nr	Mean diff.	SEM	*p*	Trial nr	Trial nr	Mean diff.	SEM	*p*	Trial nr	Trial nr	Mean diff.	SEM	*p*
1	2	0.100	0.162	0.539	1	2	0.321	0.168	0.058	1	2	0.276	0.165	0.097	1	2	0.241	0.165	0.146	1	2	0.233	0.162	0.152
	3	0.300	0.190	0.116		3	.571*	0.196	0.004		3	0.379	0.193	0.051		3	.414*	0.193	0.034		3	.567*	0.190	0.003
	4	.600*	0.230	0.010		4	.857*	0.239	0.000		4	.621*	0.234	0.009		4	.759*	0.234	0.002		4	1.067*	0.230	0.000
	5	.833*	0.242	0.001		5	1.107*	0.250	0.000		5	.862*	0.246	0.001		5	1.000*	0.246	0.000		5	1.233*	0.242	0.000
	6	.667*	0.248	0.008		6	.821*	0.257	0.002		6	1.069*	0.253	0.000		6	1.172*	0.253	0.000		6	1.167*	0.248	0.000
	7	.867*	0.251	0.001		7	1.071*	0.259	0.000		7	.690*	0.255	0.008		7	1.172*	0.255	0.000		7	1.267*	0.251	0.000
	8	.833*	0.249	0.001		8	1.143*	0.258	0.000		8	.897*	0.253	0.001		8	1.034*	0.253	0.000		8	1.367*	0.249	0.000
2	1	-0.100	0.162	0.539	2	1	-0.321	0.168	0.058	2	1	-0.276	0.165	0.097	2	1	-0.241	0.165	0.146	2	1	-0.233	0.162	0.152
	3	0.200	0.147	0.176		3	0.250	0.152	0.103		3	0.103	0.150	0.490		3	0.172	0.150	0.251		3	.333*	0.147	0.025
	4	.500*	0.196	0.012		4	.536*	0.203	0.009		4	0.345	0.199	0.086		4	.517*	0.199	0.010		4	.833*	0.196	0.000
	5	.733*	0.212	0.001		5	.786*	0.220	0.000		5	.586*	0.216	0.008		5	.759*	0.216	0.001		5	1.000*	0.212	0.000
	6	.567*	0.219	0.011		6	.500*	0.227	0.029		6	.793*	0.223	0.001		6	.931*	0.223	0.000		6	.933*	0.219	0.000
	7	.767*	0.223	0.001		7	.750*	0.231	0.001		7	0.414	0.227	0.070		7	.931*	0.227	0.000		7	1.033*	0.223	0.000
	8	.733*	0.225	0.001		8	.821*	0.233	0.001		8	.621*	0.229	0.008		8	.793*	0.229	0.001		8	1.133*	0.225	0.000
3	1	-0.300	0.190	0.116	3	1	-.571*	0.196	0.004	3	1	-0.379	0.193	0.051	3	1	-.414*	0.193	0.034	3	1	-.567*	0.190	0.003
	2	-0.200	0.147	0.176		2	-0.250	0.152	0.103		2	-0.103	0.150	0.490		2	-0.172	0.150	0.251		2	-.333*	0.147	0.025
	4	0.300	0.164	0.069		4	0.286	0.169	0.094		4	0.241	0.167	0.149		4	.345*	0.167	0.040		4	.500*	0.164	0.003
	5	.533*	0.192	0.006		5	.536*	0.198	0.008		5	.483*	0.195	0.014		5	.586*	0.195	0.003		5	.667*	0.192	0.001
	6	0.367	0.196	0.064		6	0.250	0.203	0.221		6	.690*	0.200	0.001		6	.759*	0.200	0.000		6	.600*	0.196	0.003
	7	.567*	0.198	0.005		7	.500*	0.205	0.016		7	0.310	0.202	0.126		7	.759*	0.202	0.000		7	.700*	0.198	0.001
	8	.533*	0.196	0.007		8	.571*	0.203	0.006		8	.517*	0.200	0.011		8	.621*	0.200	0.002		8	.800*	0.196	0.000
4	1	-.600*	0.230	0.010	4	1	-.857*	0.239	0.000	4	1	-.621*	0.234	0.009	4	1	-.759*	0.234	0.002	4	1	-1.067*	0.230	0.000
	2	-.500*	0.196	0.012		2	-.536*	0.203	0.009		2	-0.345	0.199	0.086		2	-.517*	0.199	0.010		2	-.833*	0.196	0.000
	3	-0.300	0.164	0.069		3	-0.286	0.169	0.094		3	-0.241	0.167	0.149		3	-.345*	0.167	0.040		3	-.500*	0.164	0.003
	5	0.233	0.161	0.149		5	0.250	0.166	0.135		5	0.241	0.164	0.142		5	0.241	0.164	0.142		5	0.167	0.161	0.302
	6	0.067	0.186	0.720		6	-0.036	0.192	0.853		6	.448*	0.189	0.019		6	.414*	0.189	0.030		6	0.100	0.186	0.591
	7	0.267	0.202	0.189		7	0.214	0.209	0.307		7	0.069	0.205	0.737		7	.414*	0.205	0.046		7	0.200	0.202	0.323

	8	0.233	0.201	0.248		8	0.286	0.208	0.172		8	0.276	0.204	0.179		8	0.276	0.204	0.179		8	0.300	0.201	0.138
	1	-.833*	0.242	0.001		1	-1.107*	0.250	0.000		1	-.862*	0.246	0.001		1	-1.000*	0.246	0.000		1	-1.233*	0.242	0.000
	2	-.733*	0.212	0.001		2	-.786*	0.220	0.000		2	-.586*	0.216	0.008		2	-.759*	0.216	0.001		2	-1.000*	0.212	0.000
	3	-.533*	0.192	0.006		3	-.536*	0.198	0.008		3	-.483*	0.195	0.014		3	-.586*	0.195	0.003		3	-.667*	0.192	0.001
5	4	-0.233	0.161	0.149	5	4	-0.250	0.166	0.135	5	4	-0.241	0.164	0.142	5	4	-0.241	0.164	0.142	5	4	-0.167	0.161	0.302
	6	-0.167	0.156	0.286		6	-0.286	0.161	0.079		6	0.207	0.158	0.194		6	0.172	0.158	0.278		6	-0.067	0.156	0.669
	7	0.033	0.169	0.844		7	-0.036	0.175	0.839		7	-0.172	0.172	0.318		7	0.172	0.172	0.318		7	0.033	0.169	0.844
	8	-8.882E-16	0.163	1.000		8	0.036	0.169	0.833		8	0.034	0.166	0.836		8	0.034	0.166	0.836		8	0.133	0.163	0.415
	1	-.667*	0.248	0.008		1	-.821*	0.257	0.002		1	-1.069*	0.253	0.000		1	-1.172*	0.253	0.000		1	-1.167*	0.248	0.000
	2	-.567*	0.219	0.011		2	-.500*	0.227	0.029		2	-.793*	0.223	0.001		2	-.931*	0.223	0.000		2	-.933*	0.219	0.000
	3	-0.367	0.196	0.064		3	-0.250	0.203	0.221		3	-.690*	0.200	0.001		3	-.759*	0.200	0.000		3	-.600*	0.196	0.003
6	4	-0.067	0.186	0.720	6	4	0.036	0.192	0.853	6	4	-.448*	0.189	0.019	6	4	-.414*	0.189	0.030	6	4	-0.100	0.186	0.591
	5	0.167	0.156	0.286		5	0.286	0.161	0.079		5	-0.207	0.158	0.194		5	-0.172	0.158	0.278		5	0.067	0.156	0.669
	7	0.200	0.142	0.162		7	0.250	0.147	0.092		7	-.379*	0.145	0.010		7	-8.882E-16	0.145	1.000		7	0.100	0.142	0.483
	8	0.167	0.154	0.282		8	.321*	0.160	0.046		8	-0.172	0.157	0.274		8	-0.138	0.157	0.381		8	0.200	0.154	0.197
	1	-.867*	0.251	0.001		1	-1.071*	0.259	0.000		1	-.690*	0.255	0.008		1	-1.172*	0.255	0.000		1	-1.267*	0.251	0.000
	2	-.767*	0.223	0.001		2	-.750*	0.231	0.001		2	-0.414	0.227	0.070		2	-.931*	0.227	0.000		2	-1.033*	0.223	0.000
	3	-.567*	0.198	0.005		3	-.500*	0.205	0.016		3	-0.310	0.202	0.126		3	-.759*	0.202	0.000		3	-.700*	0.198	0.001
7	4	-0.267	0.202	0.189	7	4	-0.214	0.209	0.307	7	4	-0.069	0.205	0.737	7	4	-.414*	0.205	0.046	7	4	-0.200	0.202	0.323
	5	-0.033	0.169	0.844		5	0.036	0.175	0.839		5	0.172	0.172	0.318		5	-0.172	0.172	0.318		5	-0.033	0.169	0.844
	6	-0.200	0.142	0.162		6	-0.250	0.147	0.092		6	.379*	0.145	0.010		6	8.882E-16	0.145	1.000		6	-0.100	0.142	0.483
	8	-0.033	0.155	0.830		8	0.071	0.161	0.657		8	0.207	0.158	0.192		8	-0.138	0.158	0.383		8	0.100	0.155	0.520
	1	-.833*	0.249	0.001		1	-1.143*	0.258	0.000		1	-.897*	0.253	0.001		1	-1.034*	0.253	0.000		1	-1.367*	0.249	0.000
	2	-.733*	0.225	0.001		2	-.821*	0.233	0.001		2	-.621*	0.229	0.008		2	-.793*	0.229	0.001		2	-1.133*	0.225	0.000
	3	-.533*	0.196	0.007		3	-.571*	0.203	0.006		3	-.517*	0.200	0.011		3	-.621*	0.200	0.002		3	-.800*	0.196	0.000
8	4	-0.233	0.201	0.248	8	4	-0.286	0.208	0.172	8	4	-0.276	0.204	0.179	8	4	-0.276	0.204	0.179	8	4	-0.300	0.201	0.138
	5	8.882E-16	0.163	1.000		5	-0.036	0.169	0.833		5	-0.034	0.166	0.836		5	-0.034	0.166	0.836		5	-0.133	0.163	0.415
	6	-0.167	0.154	0.282		6	-.321*	0.160	0.046		6	0.172	0.157	0.274		6	0.138	0.157	0.381		6	-0.200	0.154	0.197
	7	0.033	0.155	0.830		7	-0.071	0.161	0.657		7	-0.207	0.158	0.192		7	0.138	0.158	0.383		7	-0.100	0.155	0.520

Supplementary Table 2. Correlation coefficients between the scales used in the study and the magnitude of placebo hypoalgesia, along with the alpha (α) levels obtained for the Ten-Item Personality Inventory (TIPI).

		TIPI					SPSRQ		TAS
		TIPI-ES	TIPI-A	TIPI-C	TIPI-E	TIPI-O	SR	SP	
Magnitude of placebo hypoalgesia (change in pain ratings from pretest to posttest)	Correlation coefficient	τ = 0.074	τ = -0.101	τ = -0.160*	τ = 0.032	τ = 0.097	τ = 0.071	τ = -0.051	$r = 0.53$
	Statistical significance	$p = 0.323$	$p = 0.185$	$p = 0.034$	$p = 0.667$	$p = 0.200$	$p = 0.351$	$p = 0.493$	$p = 0.617$
α Cronbacha		0.743	0.518	0.741	0.741	0.417			

Note: Except for the TIPI-C scale and the number of reinforcers, none of the other scales' correlation coefficients reached significance.
Rewards = the number of rewards distributed to the participant.
Abbreviations: TIPI, Ten-Item Personality Index; ES, Emotional stability scale; A, Agreeableness scale; C, Conscientiousness scale; E, Extraversion scale; O, Openness to experience scale; SPSRQ, The Sensitivity to Punishment and Sensitivity to Reward Questionnaire; SR, Sensitivity to Rewards scale; SP, Sensitivity to Punishment scale; TAS, The Tellegen Absorption Scale.
